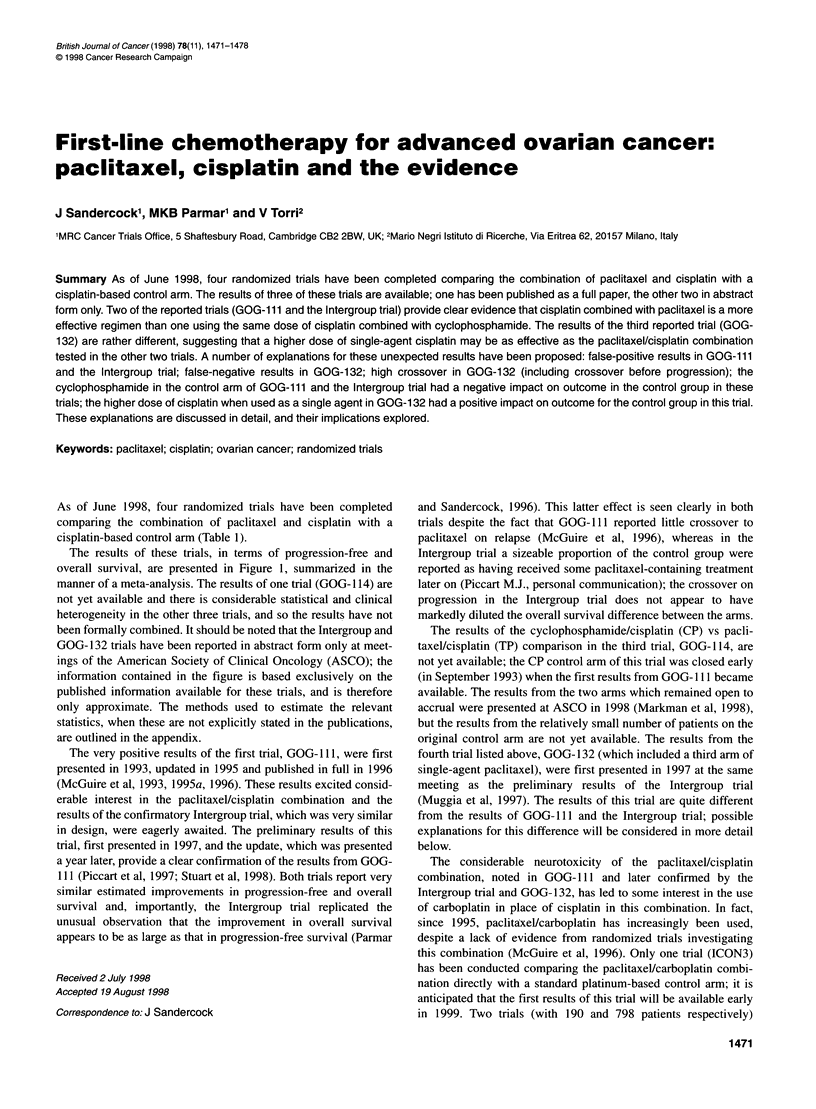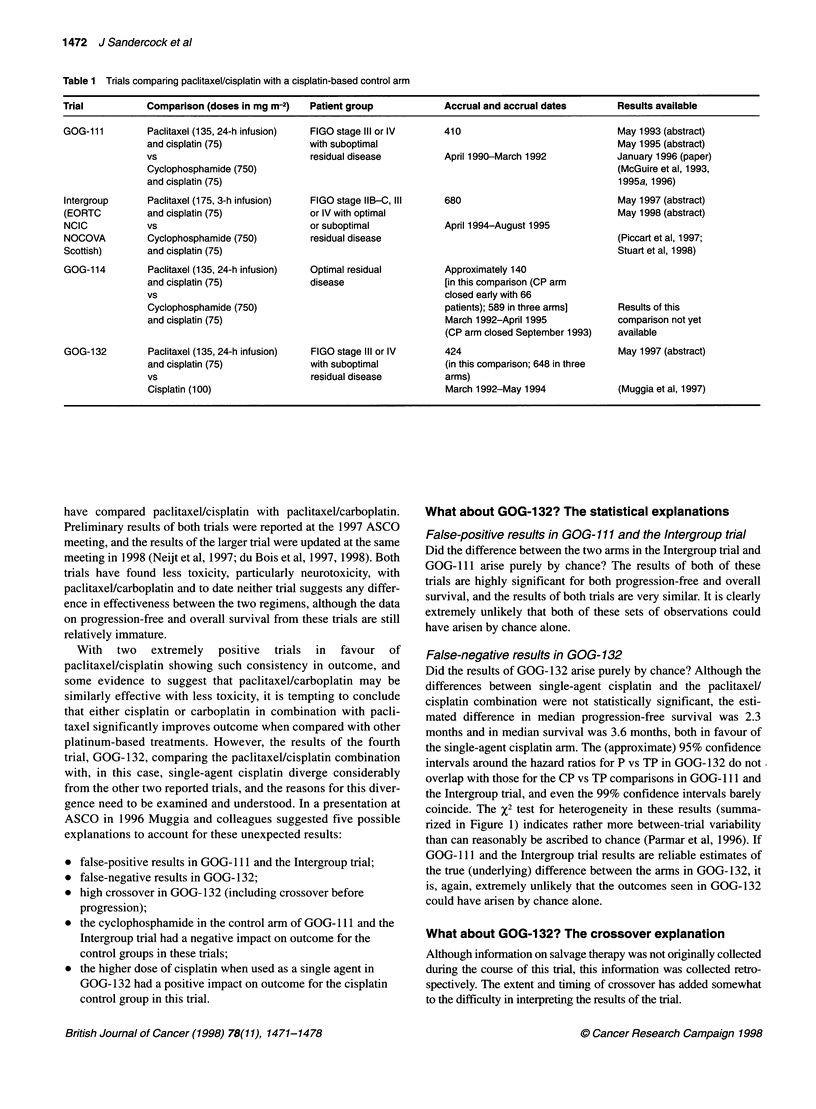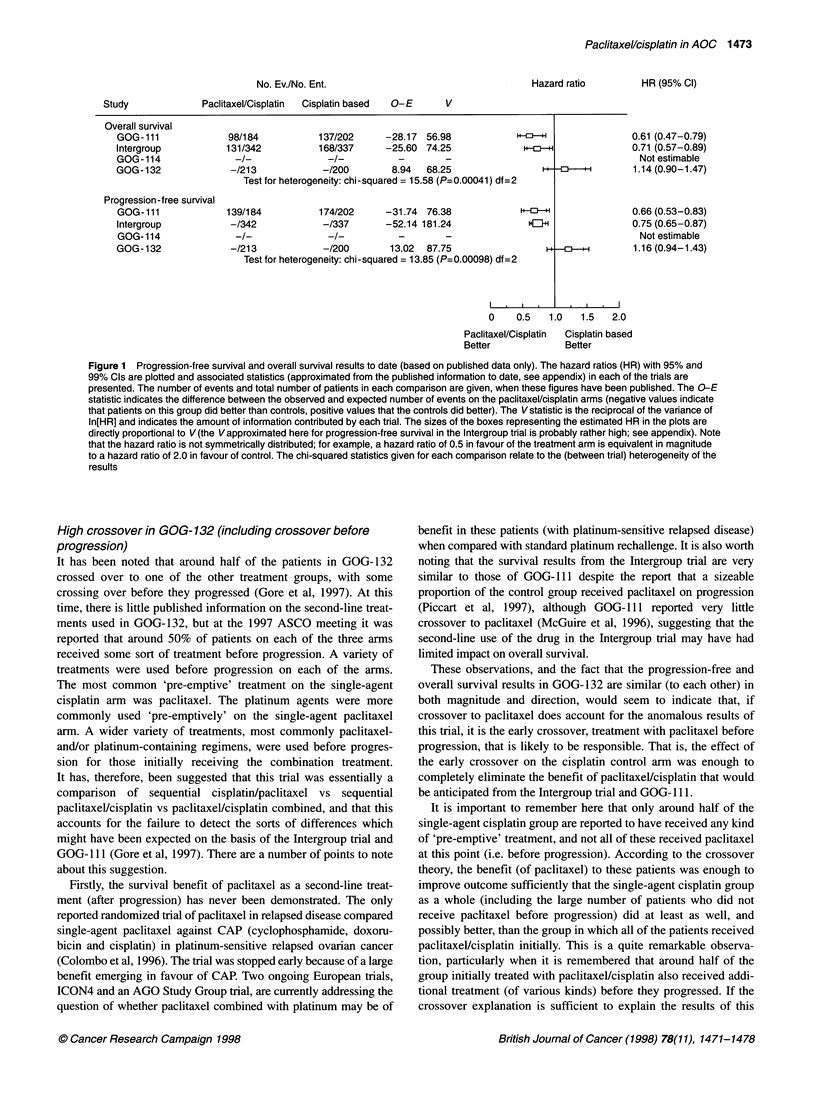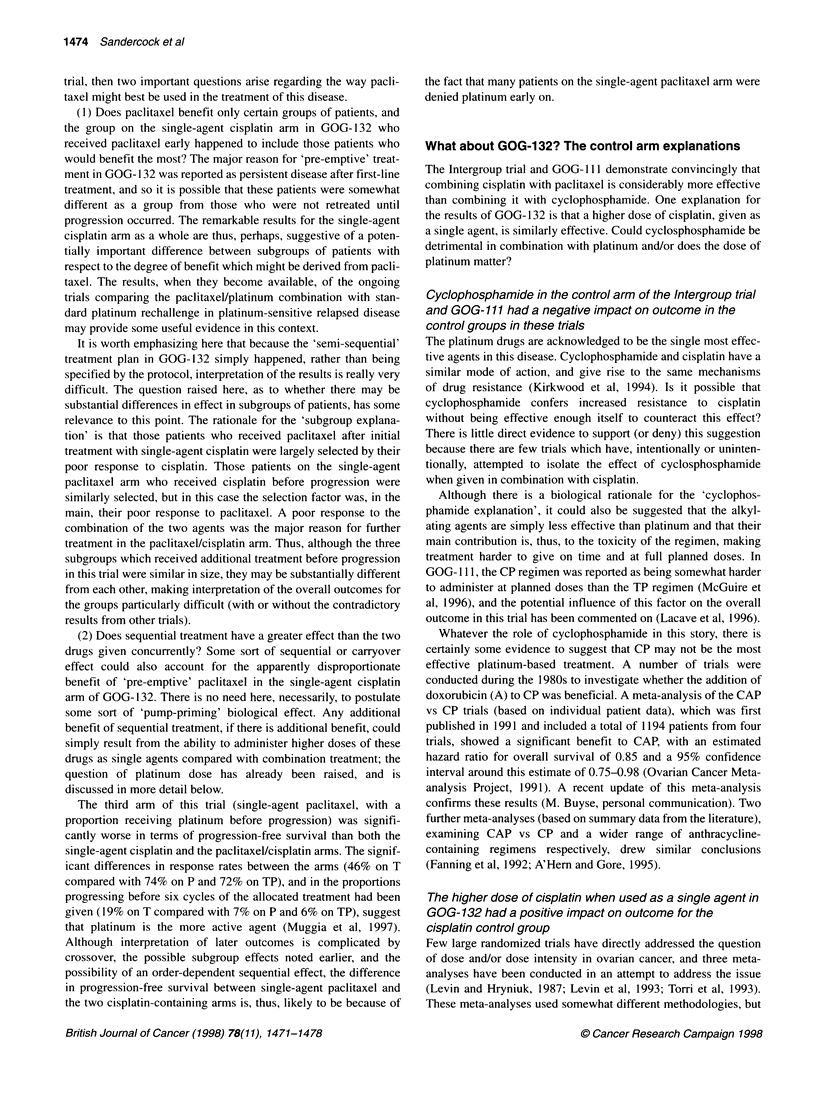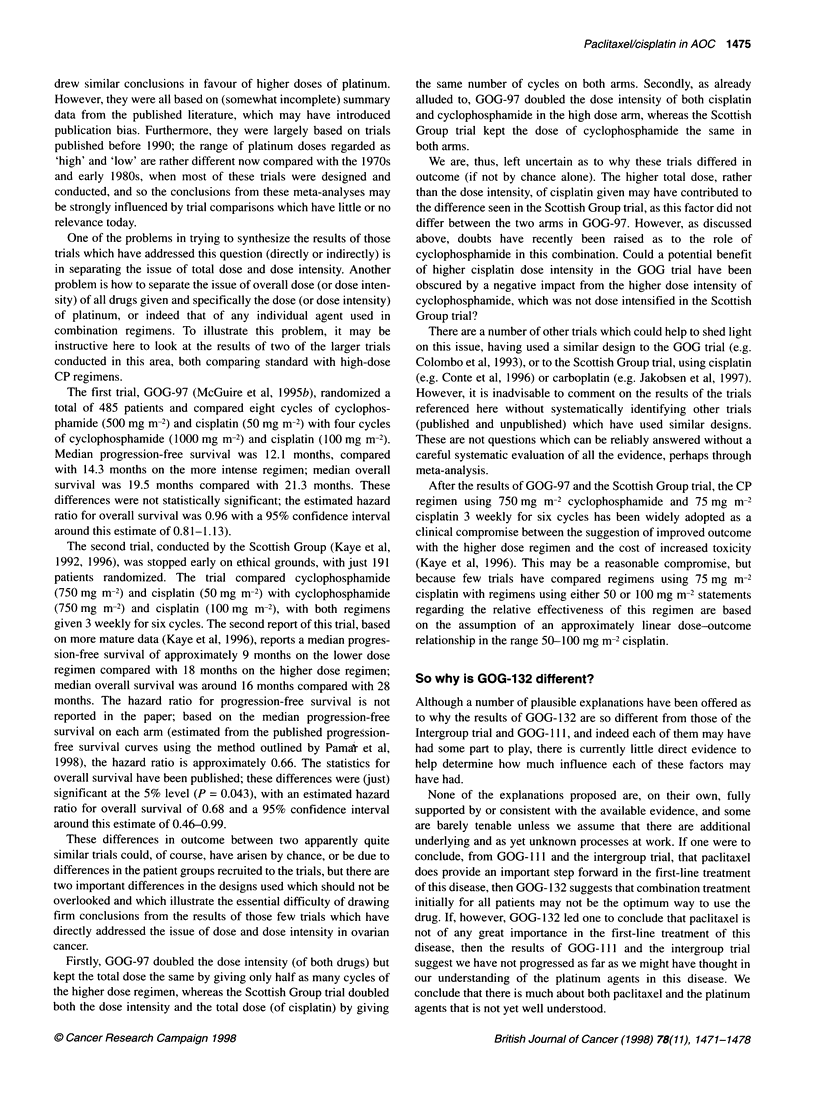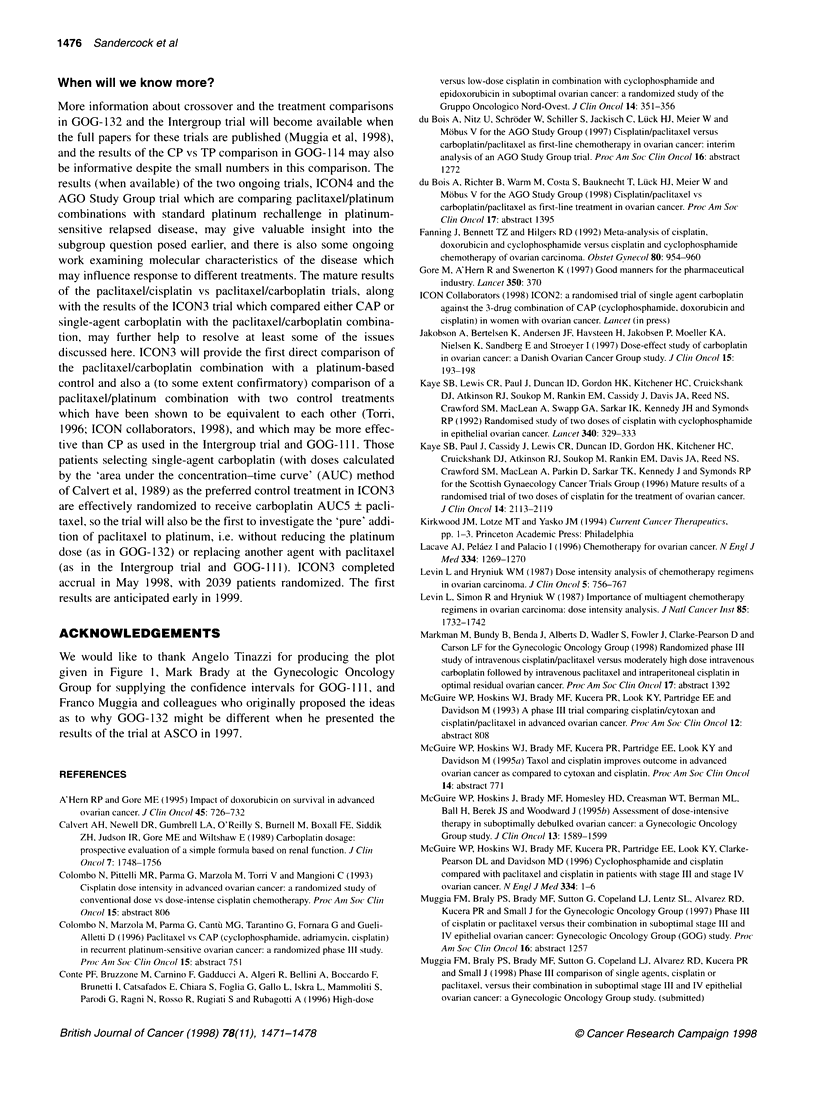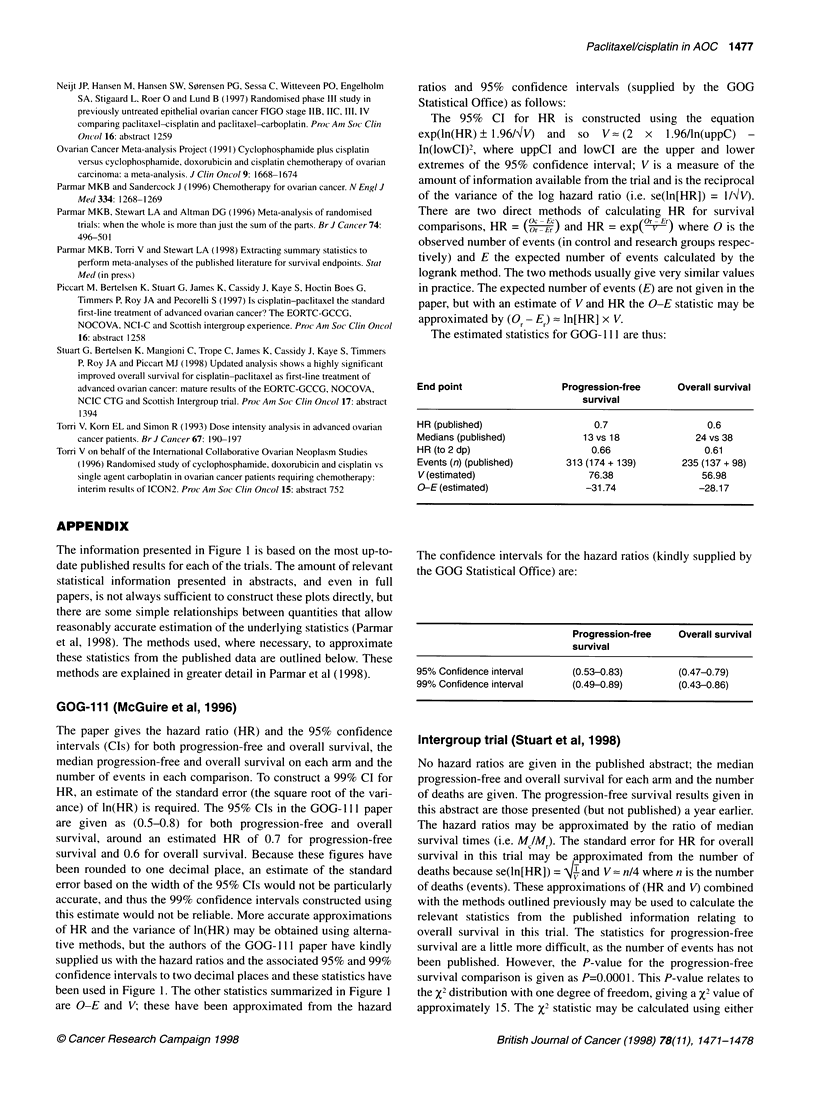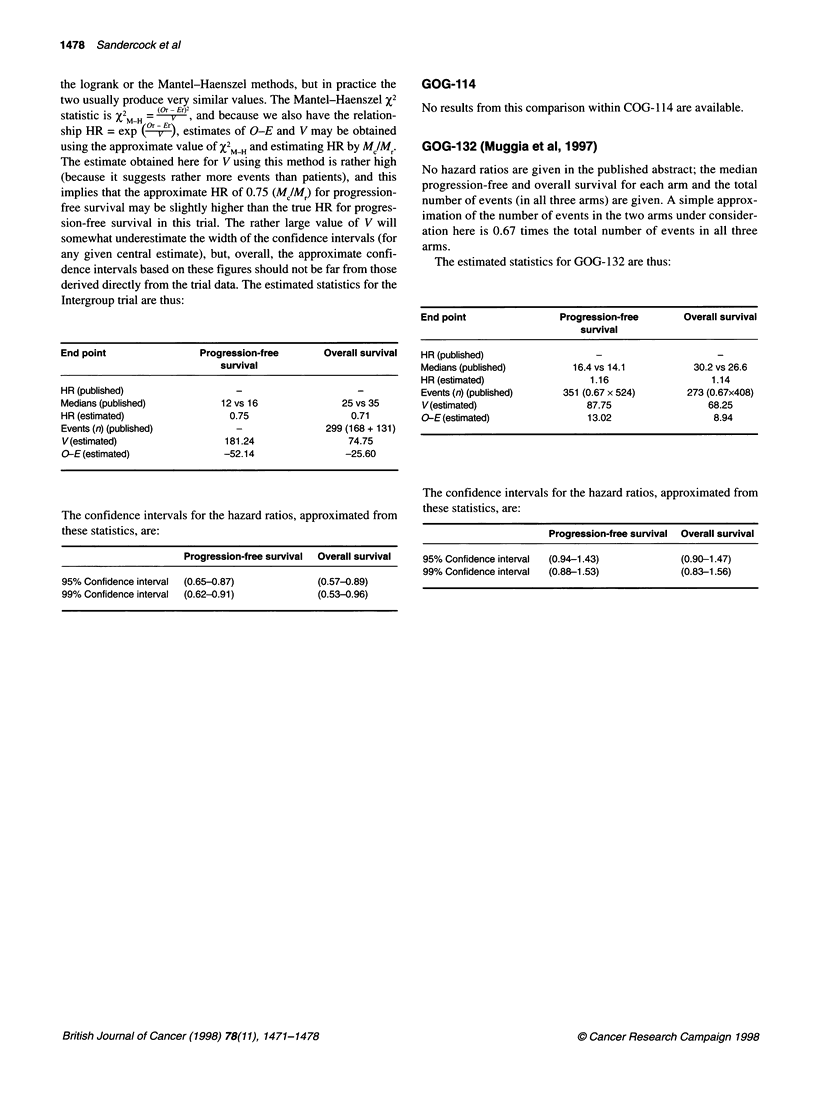# First-line chemotherapy for advanced ovarian cancer: paclitaxel, cisplatin and the evidence.

**DOI:** 10.1038/bjc.1998.709

**Published:** 1998-12

**Authors:** J. Sandercock, M. K. Parmar, V. Torri

**Affiliations:** MRC Cancer Trials Office, Cambridge, UK.

## Abstract

As of June 1998, four randomized trials have been completed comparing the combination of paclitaxel and cisplatin with a cisplatin-based control arm. The results of three of these trials are available; one has been published as a full paper, the other two in abstract form only. Two of the reported trials (GOG-111 and the Intergroup trial) provide clear evidence that cisplatin combined with paclitaxel is a more effective regimen than one using the same dose of cisplatin combined with cyclophosphamide. The results of the third reported trial (GOG-132) are rather different, suggesting that a higher dose of single-agent cisplatin may be as effective as the paclitaxel/cisplatin combination tested in the other two trials. A number of explanations for these unexpected results have been proposed: false-positive results in GOG-111 and the Intergroup trial; false-negative results in GOG-132; high crossover in GOG-132 (including crossover before progression); the cyclophosphamide in the control arm of GOG-111 and the Intergroup trial had a negative impact on outcome in the control group in these trials; the higher dose of cisplatin when used as a single agent in GOG-132 had a positive impact on outcome for the control group in this trial. These explanations are discussed in detail, and their implications explored.


British Journal of Cancer (1998) 78(11), 1471–1478
© 1998 Cancer Research Campaign

### First-line chemotherapy for advanced ovarian cancer: paclitaxel, cisplatin and the evidence

**J Sandercock[1], MKB Parmar[1] and V Torri[2]**

[1]MRC Cancer Trials Office, 5 Shaftesbury Road, Cambridge CB2 2BW, UK; [2]Mario Negri Istituto di Ricerche, Via Eritrea 62, 20157 Milano, Italy

**Summary** As of June 1998, four randomized trials have been completed comparing the combination of paclitaxel and cisplatin with a cisplatin-based control arm. The results of three of these trials are available; one has been published as a full paper, the other two in abstract form only. Two of the reported trials (GOG-111 and the Intergroup trial) provide clear evidence that cisplatin combined with paclitaxel is a more effective regimen than one using the same dose of cisplatin combined with cyclophosphamide. The results of the third reported trial (GOG-132) are rather different, suggesting that a higher dose of single-agent cisplatin may be as effective as the paclitaxel/cisplatin combination tested in the other two trials. A number of explanations for these unexpected results have been proposed: false-positive results in GOG-111 and the Intergroup trial; false-negative results in GOG-132; high crossover in GOG-132 (including crossover before progression); the cyclophosphamide in the control arm of GOG-111 and the Intergroup trial had a negative impact on outcome in the control group in these trials; the higher dose of cisplatin when used as a single agent in GOG-132 had a positive impact on outcome for the control group in this trial. These explanations are discussed in detail, and their implications explored.

**Keywords:** paclitaxel; cisplatin; ovarian cancer; randomized trials

As of June 1998, four randomized trials have been completed comparing the combination of paclitaxel and cisplatin with a cisplatin-based control arm (Table 1).

The results of these trials, in terms of progression-free and overall survival, are presented in Figure 1, summarized in the manner of a meta-analysis. The results of one trial (GOG-114) are not yet available and there is considerable statistical and clinical heterogeneity in the other three trials, and so the results have not been formally combined. It should be noted that the Intergroup and GOG-132 trials have been reported in abstract form only at meetings of the American Society of Clinical Oncology (ASCO); the information contained in the figure is based exclusively on the published information available for these trials, and is therefore only approximate. The methods used to estimate the relevant statistics, when these are not explicitly stated in the publications, are outlined in the appendix.

The very positive results of the first trial, GOG-111, were first presented in 1993, updated in 1995 and published in full in 1996 (McGuire et al, 1993, 1995*a*, 1996). These results excited considerable interest in the paclitaxel/cisplatin combination and the results of the confirmatory Intergroup trial, which was very similar in design, were eagerly awaited. The preliminary results of this trial, first presented in 1997, and the update, which was presented a year later, provide a clear confirmation of the results from GOG-111 (Piccart et al, 1997; Stuart et al, 1998). Both trials report very similar estimated improvements in progression-free and overall survival and, importantly, the Intergroup trial replicated the unusual observation that the improvement in overall survival appears to be as large as that in progression-free survival (Parmar and Sandercock, 1996). This latter effect is seen clearly in both trials despite the fact that GOG-111 reported little crossover to paclitaxel on relapse (McGuire et al, 1996), whereas in the Intergroup trial a sizeable proportion of the control group were reported as having received some paclitaxel-containing treatment later on (Piccart M.J., personal communication); the crossover on progression in the Intergroup trial does not appear to have markedly diluted the overall survival difference between the arms.

The results of the cyclophosphamide/cisplatin (CP) vs paclitaxel/cisplatin (TP) comparison in the third trial, GOG-114, are not yet available; the CP control arm of this trial was closed early (in September 1993) when the first results from GOG-111 became available. The results from the two arms which remained open to accrual were presented at ASCO in 1998 (Markman et al, 1998), but the results from the relatively small number of patients on the original control arm are not yet available. The results from the fourth trial listed above, GOG-132 (which included a third arm of single-agent paclitaxel), were first presented in 1997 at the same meeting as the preliminary results of the Intergroup trial (Muggia et al, 1997). The results of this trial are quite different from the results of GOG-111 and the Intergroup trial; possible explanations for this difference will be considered in more detail below.

The considerable neurotoxicity of the paclitaxel/cisplatin combination, noted in GOG-111 and later confirmed by the Intergroup trial and GOG-132, has led to some interest in the use of carboplatin in place of cisplatin in this combination. In fact, since 1995, paclitaxel/carboplatin has increasingly been used, despite a lack of evidence from randomized trials investigating this combination (McGuire et al, 1996). Only one trial (ICON3) has been conducted comparing the paclitaxel/carboplatin combination directly with a standard platinum-based control arm; it is anticipated that the first results of this trial will be available early in 1999. Two trials (with 190 and 798 patients respectively)

*Received 2 July 1998*
*Accepted 19 August 1998*

*Correspondence to:* J Sandercock

**Table 1** Trials comparing paclitaxel/cisplatin with a cisplatin-based control arm

| Trial | Comparison (doses in mg m$^{-2}$) | Patient group | Accrual and accrual dates | Results available |
|---|---|---|---|---|
| GOG-111 | Paclitaxel (135, 24-h infusion) and cisplatin (75) vs Cyclophosphamide (750) and cisplatin (75) | FIGO stage III or IV with suboptimal residual disease | 410<br>April 1990–March 1992 | May 1993 (abstract)<br>May 1995 (abstract)<br>January 1996 (paper)<br>(McGuire et al, 1993, 1995*a*, 1996) |
| Intergroup (EORTC NCIC NOCOVA Scottish) | Paclitaxel (175, 3-h infusion) and cisplatin (75) vs Cyclophosphamide (750) and cisplatin (75) | FIGO stage IIB–C, III or IV with optimal or suboptimal residual disease | 680<br>April 1994–August 1995 | May 1997 (abstract)<br>May 1998 (abstract)<br>(Piccart et al, 1997; Stuart et al, 1998) |
| GOG-114 | Paclitaxel (135, 24-h infusion) and cisplatin (75) vs Cyclophosphamide (750) and cisplatin (75) | Optimal residual disease | Approximately 140 [in this comparison (CP arm closed early with 66 patients); 589 in three arms]<br>March 1992–April 1995 (CP arm closed September 1993) | Results of this comparison not yet available |
| GOG-132 | Paclitaxel (135, 24-h infusion) and cisplatin (75) vs Cisplatin (100) | FIGO stage III or IV with suboptimal residual disease | 424 (in this comparison; 648 in three arms)<br>March 1992–May 1994 | May 1997 (abstract)<br>(Muggia et al, 1997) |

have compared paclitaxel/cisplatin with paclitaxel/carboplatin. Preliminary results of both trials were reported at the 1997 ASCO meeting, and the results of the larger trial were updated at the same meeting in 1998 (Neijt et al, 1997; du Bois et al, 1997, 1998). Both trials have found less toxicity, particularly neurotoxicity, with paclitaxel/carboplatin and to date neither trial suggests any difference in effectiveness between the two regimens, although the data on progression-free and overall survival from these trials are still relatively immature.

With two extremely positive trials in favour of paclitaxel/cisplatin showing such consistency in outcome, and some evidence to suggest that paclitaxel/carboplatin may be similarly effective with less toxicity, it is tempting to conclude that either cisplatin or carboplatin in combination with paclitaxel significantly improves outcome when compared with other platinum-based treatments. However, the results of the fourth trial, GOG-132, comparing the paclitaxel/cisplatin combination with, in this case, single-agent cisplatin diverge considerably from the other two reported trials, and the reasons for this divergence need to be examined and understood. In a presentation at ASCO in 1996 Muggia and colleagues suggested five possible explanations to account for these unexpected results:

- false-positive results in GOG-111 and the Intergroup trial;
- false-negative results in GOG-132;
- high crossover in GOG-132 (including crossover before progression);
- the cyclophosphamide in the control arm of GOG-111 and the Intergroup trial had a negative impact on outcome for the control groups in these trials;
- the higher dose of cisplatin when used as a single agent in GOG-132 had a positive impact on outcome for the cisplatin control group in this trial.

### What about GOG-132? The statistical explanations

### *False-positive results in GOG-111 and the Intergroup trial*

Did the difference between the two arms in the Intergroup trial and GOG-111 arise purely by chance? The results of both of these trials are highly significant for both progression-free and overall survival, and the results of both trials are very similar. It is clearly extremely unlikely that both of these sets of observations could have arisen by chance alone.

### *False-negative results in GOG-132*

Did the results of GOG-132 arise purely by chance? Although the differences between single-agent cisplatin and the paclitaxel/cisplatin combination were not statistically significant, the estimated difference in median progression-free survival was 2.3 months and in median survival was 3.6 months, both in favour of the single-agent cisplatin arm. The (approximate) 95% confidence intervals around the hazard ratios for P vs TP in GOG-132 do not overlap with those for the CP vs TP comparisons in GOG-111 and the Intergroup trial, and even the 99% confidence intervals barely coincide. The $\chi^2$ test for heterogeneity in these results (summarized in Figure 1) indicates rather more between-trial variability than can reasonably be ascribed to chance (Parmar et al, 1996). If GOG-111 and the Intergroup trial results are reliable estimates of the true (underlying) difference between the arms in GOG-132, it is, again, extremely unlikely that the outcomes seen in GOG-132 could have arisen by chance alone.

### What about GOG-132? The crossover explanation

Although information on salvage therapy was not originally collected during the course of this trial, this information was collected retrospectively. The extent and timing of crossover has added somewhat to the difficulty in interpreting the results of the trial.

 © Cancer Research Campaign 1998

| Study | No. Ev./No. Ent. Paclitaxel/Cisplatin | Cisplatin based | *O–E* | *V* | HR (95% CI) |
|---|---|---|---|---|---|
| Overall survival | | | | | |
| GOG-111 | 98/184 | 137/202 | −28.17 | 56.98 | 0.61 (0.47–0.79) |
| Intergroup | 131/342 | 168/337 | −25.60 | 74.25 | 0.71 (0.57–0.89) |
| GOG-114 | –/– | –/– | – | – | Not estimable |
| GOG-132 | –/213 | –/200 | 8.94 | 68.25 | 1.14 (0.90–1.47) |
| Test for heterogeneity: chi-squared = 15.58 (*P*=0.00041) df=2 | | | | | |
| Progression-free survival | | | | | |
| GOG-111 | 139/184 | 174/202 | −31.74 | 76.38 | 0.66 (0.53–0.83) |
| Intergroup | –/342 | –/337 | −52.14 | 181.24 | 0.75 (0.65–0.87) |
| GOG-114 | –/– | –/– | – | – | Not estimable |
| GOG-132 | –/213 | –/200 | 13.02 | 87.75 | 1.16 (0.94–1.43) |
| Test for heterogeneity: chi-squared = 13.85 (*P*=0.00098) df=2 | | | | | |

**Figure 1** Progression-free survival and overall survival results to date (based on published data only). The hazard ratios (HR) with 95% and 99% CIs are plotted and associated statistics (approximated from the published information to date, see appendix) in each of the trials are presented. The number of events and total number of patients in each comparison are given, when these figures have been published. The *O–E* statistic indicates the difference between the observed and expected number of events on the paclitaxel/cisplatin arms (negative values indicate that patients on this group did better than controls, positive values that the controls did better). The *V* statistic is the reciprocal of the variance of ln[HR] and indicates the amount of information contributed by each trial. The sizes of the boxes representing the estimated HR in the plots are directly proportional to *V* (the *V* approximated here for progression-free survival in the Intergroup trial is probably rather high; see appendix). Note that the hazard ratio is not symmetrically distributed; for example, a hazard ratio of 0.5 in favour of the treatment arm is equivalent in magnitude to a hazard ratio of 2.0 in favour of control. The chi-squared statistics given for each comparison relate to the (between trial) heterogeneity of the results

### *High crossover in GOG-132 (including crossover before progression)*

It has been noted that around half of the patients in GOG-132 crossed over to one of the other treatment groups, with some crossing over before they progressed (Gore et al, 1997). At this time, there is little published information on the second-line treatments used in GOG-132, but at the 1997 ASCO meeting it was reported that around 50% of patients on each of the three arms received some sort of treatment before progression. A variety of treatments were used before progression on each of the arms. The most common 'pre-emptive' treatment on the single-agent cisplatin arm was paclitaxel. The platinum agents were more commonly used 'pre-emptively' on the single-agent paclitaxel arm. A wider variety of treatments, most commonly paclitaxel- and/or platinum-containing regimens, were used before progression for those initially receiving the combination treatment. It has, therefore, been suggested that this trial was essentially a comparison of sequential cisplatin/paclitaxel vs sequential paclitaxel/cisplatin vs paclitaxel/cisplatin combined, and that this accounts for the failure to detect the sorts of differences which might have been expected on the basis of the Intergroup trial and GOG-111 (Gore et al, 1997). There are a number of points to note about this suggestion.

Firstly, the survival benefit of paclitaxel as a second-line treatment (after progression) has never been demonstrated. The only reported randomized trial of paclitaxel in relapsed disease compared single-agent paclitaxel against CAP (cyclophosphamide, doxorubicin and cisplatin) in platinum-sensitive relapsed ovarian cancer (Colombo et al, 1996). The trial was stopped early because of a large benefit emerging in favour of CAP. Two ongoing European trials, ICON4 and an AGO Study Group trial, are currently addressing the question of whether paclitaxel combined with platinum may be of benefit in these patients (with platinum-sensitive relapsed disease) when compared with standard platinum rechallenge. It is also worth noting that the survival results from the Intergroup trial are very similar to those of GOG-111 despite the report that a sizeable proportion of the control group received paclitaxel on progression (Piccart et al, 1997), although GOG-111 reported very little crossover to paclitaxel (McGuire et al, 1996), suggesting that the second-line use of the drug in the Intergroup trial may have had limited impact on overall survival.

These observations, and the fact that the progression-free and overall survival results in GOG-132 are similar (to each other) in both magnitude and direction, would seem to indicate that, if crossover to paclitaxel does account for the anomalous results of this trial, it is the early crossover, treatment with paclitaxel before progression, that is likely to be responsible. That is, the effect of the early crossover on the cisplatin control arm was enough to completely eliminate the benefit of paclitaxel/cisplatin that would be anticipated from the Intergroup trial and GOG-111.

It is important to remember here that only around half of the single-agent cisplatin group are reported to have received any kind of 'pre-emptive' treatment, and not all of these received paclitaxel at this point (i.e. before progression). According to the crossover theory, the benefit (of paclitaxel) to these patients was enough to improve outcome sufficiently that the single-agent cisplatin group as a whole (including the large number of patients who did not receive paclitaxel before progression) did at least as well, and possibly better, than the group in which all of the patients received paclitaxel/cisplatin initially. This is a quite remarkable observation, particularly when it is remembered that around half of the group initially treated with paclitaxel/cisplatin also received additional treatment (of various kinds) before they progressed. If the crossover explanation is sufficient to explain the results of this trial, then two important questions arise regarding the way paclitaxel might best be used in the treatment of this disease.

(1) Does paclitaxel benefit only certain groups of patients, and the group on the single-agent cisplatin arm in GOG-132 who received paclitaxel early happened to include those patients who would benefit the most? The major reason for 'pre-emptive' treatment in GOG-132 was reported as persistent disease after first-line treatment, and so it is possible that these patients were somewhat different as a group from those who were not retreated until progression occurred. The remarkable results for the single-agent cisplatin arm as a whole are thus, perhaps, suggestive of a potentially important difference between subgroups of patients with respect to the degree of benefit which might be derived from paclitaxel. The results, when they become available, of the ongoing trials comparing the paclitaxel/platinum combination with standard platinum rechallenge in platinum-sensitive relapsed disease may provide some useful evidence in this context.

It is worth emphasizing here that because the 'semi-sequential' treatment plan in GOG-132 simply happened, rather than being specified by the protocol, interpretation of the results is really very difficult. The question raised here, as to whether there may be substantial differences in effect in subgroups of patients, has some relevance to this point. The rationale for the 'subgroup explanation' is that those patients who received paclitaxel after initial treatment with single-agent cisplatin were largely selected by their poor response to cisplatin. Those patients on the single-agent paclitaxel arm who received cisplatin before progression were similarly selected, but in this case the selection factor was, in the main, their poor response to paclitaxel. A poor response to the combination of the two agents was the major reason for further treatment in the paclitaxel/cisplatin arm. Thus, although the three subgroups which received additional treatment before progression in this trial were similar in size, they may be substantially different from each other, making interpretation of the overall outcomes for the groups particularly difficult (with or without the contradictory results from other trials).

(2) Does sequential treatment have a greater effect than the two drugs given concurrently? Some sort of sequential or carryover effect could also account for the apparently disproportionate benefit of 'pre-emptive' paclitaxel in the single-agent cisplatin arm of GOG-132. There is no need here, necessarily, to postulate some sort of 'pump-priming' biological effect. Any additional benefit of sequential treatment, if there is additional benefit, could simply result from the ability to administer higher doses of these drugs as single agents compared with combination treatment; the question of platinum dose has already been raised, and is discussed in more detail below.

The third arm of this trial (single-agent paclitaxel, with a proportion receiving platinum before progression) was significantly worse in terms of progression-free survival than both the single-agent cisplatin and the paclitaxel/cisplatin arms. The significant differences in response rates between the arms (46% on T compared with 74% on P and 72% on TP), and in the proportions progressing before six cycles of the allocated treatment had been given (19% on T compared with 7% on P and 6% on TP), suggest that platinum is the more active agent (Muggia et al, 1997). Although interpretation of later outcomes is complicated by crossover, the possible subgroup effects noted earlier, and the possibility of an order-dependent sequential effect, the difference in progression-free survival between single-agent paclitaxel and the two cisplatin-containing arms is, thus, likely to be because of the fact that many patients on the single-agent paclitaxel arm were denied platinum early on.

### What about GOG-132? The control arm explanations

The Intergroup trial and GOG-111 demonstrate convincingly that combining cisplatin with paclitaxel is considerably more effective than combining it with cyclophosphamide. One explanation for the results of GOG-132 is that a higher dose of cisplatin, given as a single agent, is similarly effective. Could cyclosphosphamide be detrimental in combination with platinum and/or does the dose of platinum matter?

### *Cyclophosphamide in the control arm of the Intergroup trial and GOG-111 had a negative impact on outcome in the control groups in these trials*

The platinum drugs are acknowledged to be the single most effective agents in this disease. Cyclophosphamide and cisplatin have a similar mode of action, and give rise to the same mechanisms of drug resistance (Kirkwood et al, 1994). Is it possible that cyclophosphamide confers increased resistance to cisplatin without being effective enough itself to counteract this effect? There is little direct evidence to support (or deny) this suggestion because there are few trials which have, intentionally or unintentionally, attempted to isolate the effect of cyclosphosphamide when given in combination with cisplatin.

Although there is a biological rationale for the 'cyclophosphamide explanation', it could also be suggested that the alkylating agents are simply less effective than platinum and that their main contribution is, thus, to the toxicity of the regimen, making treatment harder to give on time and at full planned doses. In GOG-111, the CP regimen was reported as being somewhat harder to administer at planned doses than the TP regimen (McGuire et al, 1996), and the potential influence of this factor on the overall outcome in this trial has been commented on (Lacave et al, 1996).

Whatever the role of cyclophosphamide in this story, there is certainly some evidence to suggest that CP may not be the most effective platinum-based treatment. A number of trials were conducted during the 1980s to investigate whether the addition of doxorubicin (A) to CP was beneficial. A meta-analysis of the CAP vs CP trials (based on individual patient data), which was first published in 1991 and included a total of 1194 patients from four trials, showed a significant benefit to CAP, with an estimated hazard ratio for overall survival of 0.85 and a 95% confidence interval around this estimate of 0.75–0.98 (Ovarian Cancer Meta-analysis Project, 1991). A recent update of this meta-analysis confirms these results (M. Buyse, personal communication). Two further meta-analyses (based on summary data from the literature), examining CAP vs CP and a wider range of anthracycline-containing regimens respectively, drew similar conclusions (Fanning et al, 1992; A'Hern and Gore, 1995).

### *The higher dose of cisplatin when used as a single agent in GOG-132 had a positive impact on outcome for the cisplatin control group*

Few large randomized trials have directly addressed the question of dose and/or dose intensity in ovarian cancer, and three meta-analyses have been conducted in an attempt to address the issue (Levin and Hryniuk, 1987; Levin et al, 1993; Torri et al, 1993). These meta-analyses used somewhat different methodologies, but

© Cancer Research Campaign 1998

drew similar conclusions in favour of higher doses of platinum. However, they were all based on (somewhat incomplete) summary data from the published literature, which may have introduced publication bias. Furthermore, they were largely based on trials published before 1990; the range of platinum doses regarded as 'high' and 'low' are rather different now compared with the 1970s and early 1980s, when most of these trials were designed and conducted, and so the conclusions from these meta-analyses may be strongly influenced by trial comparisons which have little or no relevance today.

One of the problems in trying to synthesize the results of those trials which have addressed this question (directly or indirectly) is in separating the issue of total dose and dose intensity. Another problem is how to separate the issue of overall dose (or dose intensity) of all drugs given and specifically the dose (or dose intensity) of platinum, or indeed that of any individual agent used in combination regimens. To illustrate this problem, it may be instructive here to look at the results of two of the larger trials conducted in this area, both comparing standard with high-dose CP regimens.

The first trial, GOG-97 (McGuire et al, 1995*b*), randomized a total of 485 patients and compared eight cycles of cyclophosphamide (500 mg $m^{-2}$) and cisplatin (50 mg $m^{-2}$) with four cycles of cyclophosphamide (1000 mg $m^{-2}$) and cisplatin (100 mg $m^{-2}$). Median progression-free survival was 12.1 months, compared with 14.3 months on the more intense regimen; median overall survival was 19.5 months compared with 21.3 months. These differences were not statistically significant; the estimated hazard ratio for overall survival was 0.96 with a 95% confidence interval around this estimate of 0.81–1.13).

The second trial, conducted by the Scottish Group (Kaye et al, 1992, 1996), was stopped early on ethical grounds, with just 191 patients randomized. The trial compared cyclophosphamide (750 mg $m^{-2}$) and cisplatin (50 mg $m^{-2}$) with cyclophosphamide (750 mg $m^{-2}$) and cisplatin (100 mg $m^{-2}$), with both regimens given 3 weekly for six cycles. The second report of this trial, based on more mature data (Kaye et al, 1996), reports a median progression-free survival of approximately 9 months on the lower dose regimen compared with 18 months on the higher dose regimen; median overall survival was around 16 months compared with 28 months. The hazard ratio for progression-free survival is not reported in the paper; based on the median progression-free survival on each arm (estimated from the published progression-free survival curves using the method outlined by Pamar et al, 1998), the hazard ratio is approximately 0.66. The statistics for overall survival have been published; these differences were (just) significant at the 5% level ($P = 0.043$), with an estimated hazard ratio for overall survival of 0.68 and a 95% confidence interval around this estimate of 0.46–0.99.

These differences in outcome between two apparently quite similar trials could, of course, have arisen by chance, or be due to differences in the patient groups recruited to the trials, but there are two important differences in the designs used which should not be overlooked and which illustrate the essential difficulty of drawing firm conclusions from the results of those few trials which have directly addressed the issue of dose and dose intensity in ovarian cancer.

Firstly, GOG-97 doubled the dose intensity (of both drugs) but kept the total dose the same by giving only half as many cycles of the higher dose regimen, whereas the Scottish Group trial doubled both the dose intensity and the total dose (of cisplatin) by giving the same number of cycles on both arms. Secondly, as already alluded to, GOG-97 doubled the dose intensity of both cisplatin and cyclophosphamide in the high dose arm, whereas the Scottish Group trial kept the dose of cyclophosphamide the same in both arms.

We are, thus, left uncertain as to why these trials differed in outcome (if not by chance alone). The higher total dose, rather than the dose intensity, of cisplatin given may have contributed to the difference seen in the Scottish Group trial, as this factor did not differ between the two arms in GOG-97. However, as discussed above, doubts have recently been raised as to the role of cyclophosphamide in this combination. Could a potential benefit of higher cisplatin dose intensity in the GOG trial have been obscured by a negative impact from the higher dose intensity of cyclophosphamide, which was not dose intensified in the Scottish Group trial?

There are a number of other trials which could help to shed light on this issue, having used a similar design to the GOG trial (e.g. Colombo et al, 1993), or to the Scottish Group trial, using cisplatin (e.g. Conte et al, 1996) or carboplatin (e.g. Jakobsen et al, 1997). However, it is inadvisable to comment on the results of the trials referenced here without systematically identifying other trials (published and unpublished) which have used similar designs. These are not questions which can be reliably answered without a careful systematic evaluation of all the evidence, perhaps through meta-analysis.

After the results of GOG-97 and the Scottish Group trial, the CP regimen using 750 mg $m^{-2}$ cyclophosphamide and 75 mg $m^{-2}$ cisplatin 3 weekly for six cycles has been widely adopted as a clinical compromise between the suggestion of improved outcome with the higher dose regimen and the cost of increased toxicity (Kaye et al, 1996). This may be a reasonable compromise, but because few trials have compared regimens using 75 mg $m^{-2}$ cisplatin with regimens using either 50 or 100 mg $m^{-2}$ statements regarding the relative effectiveness of this regimen are based on the assumption of an approximately linear dose–outcome relationship in the range 50–100 mg $m^{-2}$ cisplatin.

### So why is GOG-132 different?

Although a number of plausible explanations have been offered as to why the results of GOG-132 are so different from those of the Intergroup trial and GOG-111, and indeed each of them may have had some part to play, there is currently little direct evidence to help determine how much influence each of these factors may have had.

None of the explanations proposed are, on their own, fully supported by or consistent with the available evidence, and some are barely tenable unless we assume that there are additional underlying and as yet unknown processes at work. If one were to conclude, from GOG-111 and the intergroup trial, that paclitaxel does provide an important step forward in the first-line treatment of this disease, then GOG-132 suggests that combination treatment initially for all patients may not be the optimum way to use the drug. If, however, GOG-132 led one to conclude that paclitaxel is not of any great importance in the first-line treatment of this disease, then the results of GOG-111 and the intergroup trial suggest we have not progressed as far as we might have thought in our understanding of the platinum agents in this disease. We conclude that there is much about both paclitaxel and the platinum agents that is not yet well understood.

© Cancer Research Campaign 1998

### When will we know more?

More information about crossover and the treatment comparisons in GOG-132 and the Intergroup trial will become available when the full papers for these trials are published (Muggia et al, 1998), and the results of the CP vs TP comparison in GOG-114 may also be informative despite the small numbers in this comparison. The results (when available) of the two ongoing trials, ICON4 and the AGO Study Group trial which are comparing paclitaxel/platinum combinations with standard platinum rechallenge in platinum-sensitive relapsed disease, may give valuable insight into the subgroup question posed earlier, and there is also some ongoing work examining molecular characteristics of the disease which may influence response to different treatments. The mature results of the paclitaxel/cisplatin vs paclitaxel/carboplatin trials, along with the results of the ICON3 trial which compared either CAP or single-agent carboplatin with the paclitaxel/carboplatin combination, may further help to resolve at least some of the issues discussed here. ICON3 will provide the first direct comparison of the paclitaxel/carboplatin combination with a platinum-based control and also a (to some extent confirmatory) comparison of a paclitaxel/platinum combination with two control treatments which have been shown to be equivalent to each other (Torri, 1996; ICON collaborators, 1998), and which may be more effective than CP as used in the Intergroup trial and GOG-111. Those patients selecting single-agent carboplatin (with doses calculated by the 'area under the concentration–time curve' (AUC) method of Calvert et al, 1989) as the preferred control treatment in ICON3 are effectively randomized to receive carboplatin AUC5 ± paclitaxel, so the trial will also be the first to investigate the 'pure' addition of paclitaxel to platinum, i.e. without reducing the platinum dose (as in GOG-132) or replacing another agent with paclitaxel (as in the Intergroup trial and GOG-111). ICON3 completed accrual in May 1998, with 2039 patients randomized. The first results are anticipated early in 1999.

### ACKNOWLEDGEMENTS

We would like to thank Angelo Tinazzi for producing the plot given in Figure 1, Mark Brady at the Gynecologic Oncology Group for supplying the confidence intervals for GOG-111, and Franco Muggia and colleagues who originally proposed the ideas as to why GOG-132 might be different when he presented the results of the trial at ASCO in 1997.

### REFERENCES

A'Hern RP and Gore ME (1995) Impact of doxorubicin on survival in advanced ovarian cancer. *J Clin Oncol* **45**: 726–732

Calvert AH, Newell DR, Gumbrell LA, O'Reilly S, Burnell M, Boxall FE, Siddik ZH, Judson IR, Gore ME and Wiltshaw E (1989) Carboplatin dosage: prospective evaluation of a simple formula based on renal function. *J Clin Oncol* **7**: 1748–1756

Colombo N, Pittelli MR, Parma G, Marzola M, Torri V and Mangioni C (1993) Cisplatin dose intensity in advanced ovarian cancer: a randomized study of conventional dose vs dose-intense cisplatin chemotherapy. *Proc Am Soc Clin Oncol* **15**: abstract 806

Colombo N, Marzola M, Parma G, Cantù MG, Tarantino G, Fornara G and Gueli-Alletti D (1996) Paclitaxel vs CAP (cyclophosphamide, adriamycin, cisplatin) in recurrent platinum-sensitive ovarian cancer: a randomized phase III study. *Proc Am Soc Clin Oncol* **15**: abstract 751

Conte PF, Bruzzone M, Carnino F, Gadducci A, Algeri R, Bellini A, Boccardo F, Brunetti I, Catsafados E, Chiara S, Foglia G, Gallo L, Iskra L, Mammoliti S, Parodi G, Ragni N, Rosso R, Rugiati S and Rubagotti A (1996) High-dose versus low-dose cisplatin in combination with cyclophosphamide and epidoxorubicin in suboptimal ovarian cancer: a randomized study of the Gruppo Oncologico Nord-Ovest. *J Clin Oncol* **14**: 351–356

du Bois A, Nitz U, Schröder W, Schiller S, Jackisch C, Lück HJ, Meier W and Möbus V for the AGO Study Group (1997) Cisplatin/paclitaxel versus carboplatin/paclitaxel as first-line chemotherapy in ovarian cancer: interim analysis of an AGO Study Group trial. *Proc Am Soc Clin Oncol* **16**: abstract 1272

du Bois A, Richter B, Warm M, Costa S, Bauknecht T, Lück HJ, Meier W and Möbus V for the AGO Study Group (1998) Cisplatin/paclitaxel vs carboplatin/paclitaxel as first-line treatment in ovarian cancer. *Proc Am Soc Clin Oncol* **17**: abstract 1395

Fanning J, Bennett TZ and Hilgers RD (1992) Meta-analysis of cisplatin, doxorubicin and cyclophosphamide versus cisplatin and cyclophosphamide chemotherapy of ovarian carcinoma. *Obstet Gynecol* **80**: 954–960

Gore M, A'Hern R and Swenerton K (1997) Good manners for the pharmaceutical industry. *Lancet* **350**: 370

ICON Collaborators (1998) ICON2: a randomised trial of single agent carboplatin against the 3-drug combination of CAP (cyclophosphamide, doxorubicin and cisplatin) in women with ovarian cancer. *Lancet* (in press)

Jakobson A, Bertelsen K, Andersen JF, Havsteen H, Jakobsen P, Moeller KA, Nielsen K, Sandberg E and Stroeyer I (1997) Dose-effect study of carboplatin in ovarian cancer: a Danish Ovarian Cancer Group study. *J Clin Oncol* **15**: 193–198

Kaye SB, Lewis CR, Paul J, Duncan ID, Gordon HK, Kitchener HC, Cruickshank DJ, Atkinson RJ, Soukop M, Rankin EM, Cassidy J, Davis JA, Reed NS, Crawford SM, MacLean A, Swapp GA, Sarkar IK, Kennedy JH and Symonds RP (1992) Randomised study of two doses of cisplatin with cyclophosphamide in epithelial ovarian cancer. *Lancet* **340**: 329–333

Kaye SB, Paul J, Cassidy J, Lewis CR, Duncan ID, Gordon HK, Kitchener HC, Cruickshank DJ, Atkinson RJ, Soukop M, Rankin EM, Davis JA, Reed NS, Crawford SM, MacLean A, Parkin D, Sarkar TK, Kennedy J and Symonds RP for the Scottish Gynaecology Cancer Trials Group (1996) Mature results of a randomised trial of two doses of cisplatin for the treatment of ovarian cancer. *J Clin Oncol* **14**: 2113–2119

Kirkwood JM, Lotze MT and Yasko JM (1994) *Current Cancer Therapeutics*, pp. 1–3. Princeton Academic Press: Philadelphia

Lacave AJ, Peláez I and Palacio I (1996) Chemotherapy for ovarian cancer. *N Engl J Med* **334**: 1269–1270

Levin L and Hryniuk WM (1987) Dose intensity analysis of chemotherapy regimens in ovarian carcinoma. *J Clin Oncol* **5**: 756–767

Levin L, Simon R and Hryniuk W (1987) Importance of multiagent chemotherapy regimens in ovarian carcinoma: dose intensity analysis. *J Natl Cancer Inst* **85**: 1732–1742

Markman M, Bundy B, Benda J, Alberts D, Wadler S, Fowler J, Clarke-Pearson D and Carson LF for the Gynecologic Oncology Group (1998) Randomized phase III study of intravenous cisplatin/paclitaxel versus moderately high dose intravenous carboplatin followed by intravenous paclitaxel and intraperitoneal cisplatin in optimal residual ovarian cancer. *Proc Am Soc Clin Oncol* **17**: abstract 1392

McGuire WP, Hoskins WJ, Brady MF, Kucera PR, Look KY, Partridge EE and Davidson M (1993) A phase III trial comparing cisplatin/cytoxan and cisplatin/paclitaxel in advanced ovarian cancer. *Proc Am Soc Clin Oncol* **12**: abstract 808

McGuire WP, Hoskins WJ, Brady MF, Kucera PR, Partridge EE, Look KY and Davidson M (1995*a*) Taxol and cisplatin improves outcome in advanced ovarian cancer as compared to cytoxan and cisplatin. *Proc Am Soc Clin Oncol* **14**: abstract 771

McGuire WP, Hoskins J, Brady MF, Homesley HD, Creasman WT, Berman ML, Ball H, Berek JS and Woodward J (1995*b*) Assessment of dose-intensive therapy in suboptimally debulked ovarian cancer: a Gynecologic Oncology Group study. *J Clin Oncol* **13**: 1589–1599

McGuire WP, Hoskins WJ, Brady MF, Kucera PR, Partridge EE, Look KY, Clarke-Pearson DL and Davidson MD (1996) Cyclophosphamide and cisplatin compared with paclitaxel and cisplatin in patients with stage III and stage IV ovarian cancer. *N Engl J Med* **334**: 1–6

Muggia FM, Braly PS, Brady MF, Sutton G, Copeland LJ, Lentz SL, Alvarez RD, Kucera PR and Small J for the Gynecologic Oncology Group (1997) Phase III of cisplatin or paclitaxel versus their combination in suboptimal stage III and IV epithelial ovarian cancer: Gynecologic Oncology Group (GOG) study. *Proc Am Soc Clin Oncol* **16**: abstract 1257

Muggia FM, Braly PS, Brady MF, Sutton G, Copeland LJ, Alvarez RD, Kucera PR and Small J (1998) Phase III comparison of single agents, cisplatin or paclitaxel, versus their combination in suboptimal stage III and IV epithelial ovarian cancer: a Gynecologic Oncology Group study. (submitted)

 © Cancer Research Campaign 1998

Neijt JP, Hansen M, Hansen SW, Sørensen PG, Sessa C, Witteveen PO, Engelholm SA, Stigaard L, Roer O and Lund B (1997) Randomised phase III study in previously untreated epithelial ovarian cancer FIGO stage IIB, IIC, III, IV comparing paclitaxel–cisplatin and paclitaxel–carboplatin. *Proc Am Soc Clin Oncol* **16**: abstract 1259

Ovarian Cancer Meta-analysis Project (1991) Cyclophosphamide plus cisplatin versus cyclophosphamide, doxorubicin and cisplatin chemotherapy of ovarian carcinoma: a meta-analysis. *J Clin Oncol* **9**: 1668–1674

Parmar MKB and Sandercock J (1996) Chemotherapy for ovarian cancer. *N Engl J Med* **334**: 1268–1269

Parmar MKB, Stewart LA and Altman DG (1996) Meta-analysis of randomised trials: when the whole is more than just the sum of the parts. *Br J Cancer* **74**: 496–501

Parmar MKB, Torri V and Stewart LA (1998) Extracting summary statistics to perform meta-analyses of the published literature for survival endpoints. *Stat Med* (in press)

Piccart M, Bertelsen K, Stuart G, James K, Cassidy J, Kaye S, Hoctin Boes G, Timmers P, Roy JA and Pecorelli S (1997) Is cisplatin–paclitaxel the standard first-line treatment of advanced ovarian cancer? The EORTC-GCCG, NOCOVA, NCI-C and Scottish intergroup experience. *Proc Am Soc Clin Oncol* **16**: abstract 1258

Stuart G, Bertelsen K, Mangioni C, Trope C, James K, Cassidy J, Kaye S, Timmers P, Roy JA and Piccart MJ (1998) Updated analysis shows a highly significant improved overall survival for cisplatin–paclitaxel as first-line treatment of advanced ovarian cancer: mature results of the EORTC-GCCG, NOCOVA, NCIC CTG and Scottish Intergroup trial. *Proc Am Soc Clin Oncol* **17**: abstract 1394

Torri V, Korn EL and Simon R (1993) Dose intensity analysis in advanced ovarian cancer patients. *Br J Cancer* **67**: 190–197

Torri V on behalf of the International Collaborative Ovarian Neoplasm Studies (1996) Randomised study of cyclophosphamide, doxorubicin and cisplatin vs single agent carboplatin in ovarian cancer patients requiring chemotherapy: interim results of ICON2. *Proc Am Soc Clin Oncol* **15**: abstract 752

### APPENDIX

The information presented in Figure 1 is based on the most up-to-date published results for each of the trials. The amount of relevant statistical information presented in abstracts, and even in full papers, is not always sufficient to construct these plots directly, but there are some simple relationships between quantities that allow reasonably accurate estimation of the underlying statistics (Parmar et al, 1998). The methods used, where necessary, to approximate these statistics from the published data are outlined below. These methods are explained in greater detail in Parmar et al (1998).

### GOG-111 (McGuire et al, 1996)

The paper gives the hazard ratio (HR) and the 95% confidence intervals (CIs) for both progression-free and overall survival, the median progression-free and overall survival on each arm and the number of events in each comparison. To construct a 99% CI for HR, an estimate of the standard error (the square root of the variance) of ln(HR) is required. The 95% CIs in the GOG-111 paper are given as (0.5–0.8) for both progression-free and overall survival, around an estimated HR of 0.7 for progression-free survival and 0.6 for overall survival. Because these figures have been rounded to one decimal place, an estimate of the standard error based on the width of the 95% CIs would not be particularly accurate, and thus the 99% confidence intervals constructed using this estimate would not be reliable. More accurate approximations of HR and the variance of ln(HR) may be obtained using alternative methods, but the authors of the GOG-111 paper have kindly supplied us with the hazard ratios and the associated 95% and 99% confidence intervals to two decimal places and these statistics have been used in Figure 1. The other statistics summarized in Figure 1 are *O–E* and *V*; these have been approximated from the hazard ratios and 95% confidence intervals (supplied by the GOG Statistical Office) as follows:

The 95% CI for HR is constructed using the equation $\exp(\ln(\mathrm{HR}) \pm 1.96/\sqrt{V})$ and so $V \approx (2 \times 1.96/\ln(\mathrm{uppC}) - \ln(\mathrm{lowCI})^2$, where uppCI and lowCI are the upper and lower extremes of the 95% confidence interval; *V* is a measure of the amount of information available from the trial and is the reciprocal of the variance of the log hazard ratio (i.e. $\mathrm{se}(\ln[\mathrm{HR}]) = 1/\sqrt{V}$). There are two direct methods of calculating HR for survival comparisons, $\mathrm{HR} = \left(\frac{Oc - Ec}{Or - Er}\right)$ and $\mathrm{HR} = \exp\left(\frac{Or - Er}{V}\right)$ where *O* is the observed number of events (in control and research groups respectively) and *E* the expected number of events calculated by the logrank method. The two methods usually give very similar values in practice. The expected number of events (*E*) are not given in the paper, but with an estimate of *V* and HR the *O–E* statistic may be approximated by $(O_r - E_r) \approx \ln[\mathrm{HR}] \times V$.

The estimated statistics for GOG-111 are thus:

| End point | Progression-free survival | Overall survival |
|---|---|---|
| HR (published) | 0.7 | 0.6 |
| Medians (published) | 13 vs 18 | 24 vs 38 |
| HR (to 2 dp) | 0.66 | 0.61 |
| Events (*n*) (published) | 313 (174 + 139) | 235 (137 + 98) |
| *V* (estimated) | 76.38 | 56.98 |
| *O–E* (estimated) | –31.74 | –28.17 |

The confidence intervals for the hazard ratios (kindly supplied by the GOG Statistical Office) are:

| | Progression-free survival | Overall survival |
|---|---|---|
| 95% Confidence interval | (0.53–0.83) | (0.47–0.79) |
| 99% Confidence interval | (0.49–0.89) | (0.43–0.86) |

### Intergroup trial (Stuart et al, 1998)

No hazard ratios are given in the published abstract; the median progression-free and overall survival for each arm and the number of deaths are given. The progression-free survival results given in this abstract are those presented (but not published) a year earlier. The hazard ratios may be approximated by the ratio of median survival times (i.e. $M_c/M_r$). The standard error for HR for overall survival in this trial may be approximated from the number of deaths because $\mathrm{se}(\ln[\mathrm{HR}]) = \sqrt{\frac{1}{V}}$ and $V \approx n/4$ where *n* is the number of deaths (events). These approximations of (HR and *V*) combined with the methods outlined previously may be used to calculate the relevant statistics from the published information relating to overall survival in this trial. The statistics for progression-free survival are a little more difficult, as the number of events has not been published. However, the *P*-value for the progression-free survival comparison is given as $P$=0.0001. This *P*-value relates to the $\chi^2$ distribution with one degree of freedom, giving a $\chi^2$ value of approximately 15. The $\chi^2$ statistic may be calculated using either

© Cancer Research Campaign 1998 

the logrank or the Mantel–Haenszel methods, but in practice the two usually produce very similar values. The Mantel–Haenszel $\chi^2$ statistic is $\chi^2_{M-H} = \frac{(Or - Er)^2}{V}$, and because we also have the relationship HR = exp $(\frac{Or - Er}{V})$, estimates of *O–E* and *V* may be obtained using the approximate value of $\chi^2_{M-H}$ and estimating HR by $M_c/M_r$. The estimate obtained here for *V* using this method is rather high (because it suggests rather more events than patients), and this implies that the approximate HR of 0.75 ($M_c/M_r$) for progression-free survival may be slightly higher than the true HR for progression-free survival in this trial. The rather large value of *V* will somewhat underestimate the width of the confidence intervals (for any given central estimate), but, overall, the approximate confidence intervals based on these figures should not be far from those derived directly from the trial data. The estimated statistics for the Intergroup trial are thus:

| End point | Progression-free survival | Overall survival |
|---|---|---|
| HR (published) | – | – |
| Medians (published) | 12 vs 16 | 25 vs 35 |
| HR (estimated) | 0.75 | 0.71 |
| Events (*n*) (published) | – | 299 (168 + 131) |
| *V* (estimated) | 181.24 | 74.75 |
| *O–E* (estimated) | –52.14 | –25.60 |

The confidence intervals for the hazard ratios, approximated from these statistics, are:

| | Progression-free survival | Overall survival |
|---|---|---|
| 95% Confidence interval | (0.65–0.87) | (0.57–0.89) |
| 99% Confidence interval | (0.62–0.91) | (0.53–0.96) |

### GOG-114

No results from this comparison within COG-114 are available.

### GOG-132 (Muggia et al, 1997)

No hazard ratios are given in the published abstract; the median progression-free and overall survival for each arm and the total number of events (in all three arms) are given. A simple approximation of the number of events in the two arms under consideration here is 0.67 times the total number of events in all three arms.

The estimated statistics for GOG-132 are thus:

| End point | Progression-free survival | Overall survival |
|---|---|---|
| HR (published) | – | – |
| Medians (published) | 16.4 vs 14.1 | 30.2 vs 26.6 |
| HR (estimated) | 1.16 | 1.14 |
| Events (*n*) (published) | 351 (0.67 x 524) | 273 (0.67x408) |
| *V* (estimated) | 87.75 | 68.25 |
| *O–E* (estimated) | 13.02 | 8.94 |

The confidence intervals for the hazard ratios, approximated from these statistics, are:

| | Progression-free survival | Overall survival |
|---|---|---|
| 95% Confidence interval | (0.94–1.43) | (0.90–1.47) |
| 99% Confidence interval | (0.88–1.53) | (0.83–1.56) |

© Cancer Research Campaign 1998